# Atypical Refractory Macular Edema: Are We Missing Something?

**DOI:** 10.18502/jovr.v17i3.11584

**Published:** 2022-08-15

**Authors:** Chaitra Jayadev, Ishank Gupta, Santosh Gopi Krishna Gadde

**Affiliations:** ^1^Department of Vitreoretina, Narayana Nethralaya Eye Institute, Bangalore, India

**Keywords:** Atypical Macular Edema, Multiple Myeloma, Paraproteinemic Maculopathy, Serous Macular Detachment

## Abstract

**Purpose:**

To report a case of bilateral refractory macular edema in a diabetic macular edema in a diabetic with an underlying systemic illness.

**Case Report:**

A 65-year-old male presented with the symptom of blurred vision in both eyes for three months. He was a known diabetic patient and was also hypertensive for the last 10 years. The corrected distance visual acuity was 20/120 in the right eye and 20/80 in the left eye. Fundus examination revealed multiple deep and superficial retinal hemorrhages, cystoid macular edema, and serous macular detachment in both eyes. With a diagnosis of diabetic macular edema in both eyes, the patient was treated with multiple intravitreal injections of anti-vascular endothelial growth factor and steroids. Since he did not show a favorable response, the patient was further investigated and diagnosed with multiple myeloma. After undergoing treatment for the same, the patient was seen a year later and noted to have significant resolution of the macular edema and subretinal fluid in both eyes.

**Conclusion:**

In patients who suffer with atypical macular edema that is resistant to conventional treatment, it is imperative to look for underlying systemic illnesses such as immunoproliferative disorders and hematologic malignancies.

##  INTRODUCTION

Diabetic macular edema (DME), characterized as a thickening of the retina involving or approaching the center of the macula, is a common cause of vision loss in patients with diabetes mellitus.^[1]^ DME has a complex pathogenesis and course and needs a multidisciplinary approach for systemic control and local disease management.^[2]^ Typically, the morphological patterns of non-tractional DME, as described on optical coherence tomography (OCT), consist of cystoid macular edema (CME), sponge-like or diffuse edema, and serous retinal detachment.^[3]^

Other causes of serous macular detachment (SMD) and macular edema include chronic central serous chorioretinopathy with or without an associated choroidal neovascularization, exudative age-related macular degeneration, polypoidal choroidal vasculopathy, retinal vein occlusions, Harada's disease, hypertensive retinopathy, retinal macroaneurysms, and optic disc pit maculopathy.^[4]^ Blood dyscrasia and paraproteinemia are rare causes of neurosensory detachments. Multiple myeloma accounts for 10% of all hematological malignancies and funduscopic abnormalities are seen in approximately 30–40% of patients with immunogammopathy.^[5]^ We report a diabetic patient with atypical macular edema, nonresponsive to conventional treatment, who was investigated and diagnosed to have an underlying life-threatening systemic pathology.

**Figure 1:**
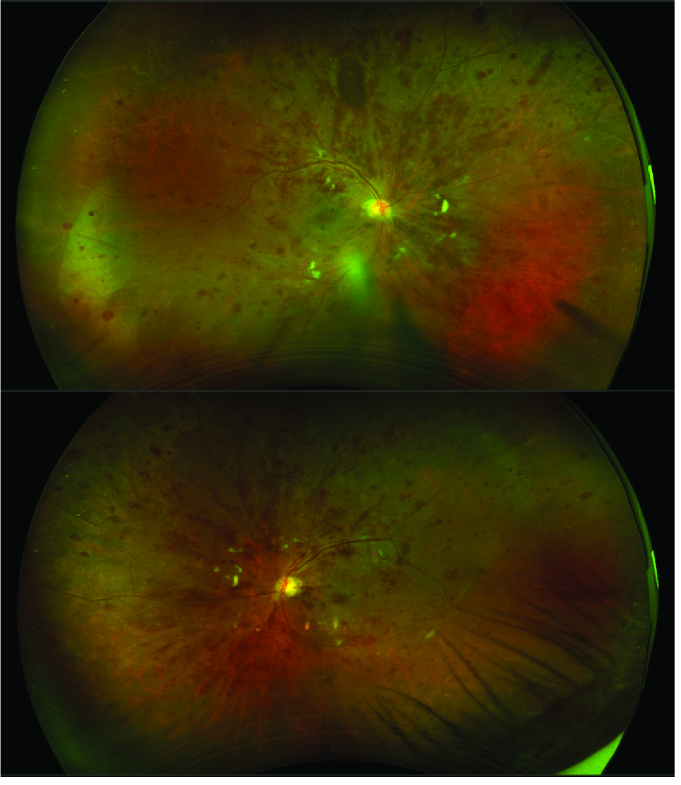
Color fundus images of the right (above) and left (below) eyes, showing a blunted foveal reflex, scattered hemorrhages, and dilated tortuous vessels.

**Figure 2:**
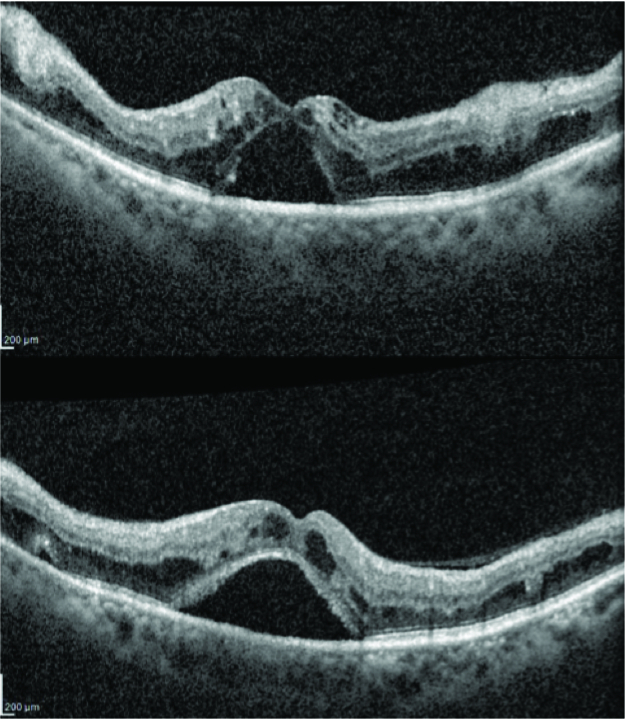
Optical coherence tomography of the right (above) and left (below) eyes at presentation showing cystoid macular edema and serous macular detachment.

**Figure 3:**
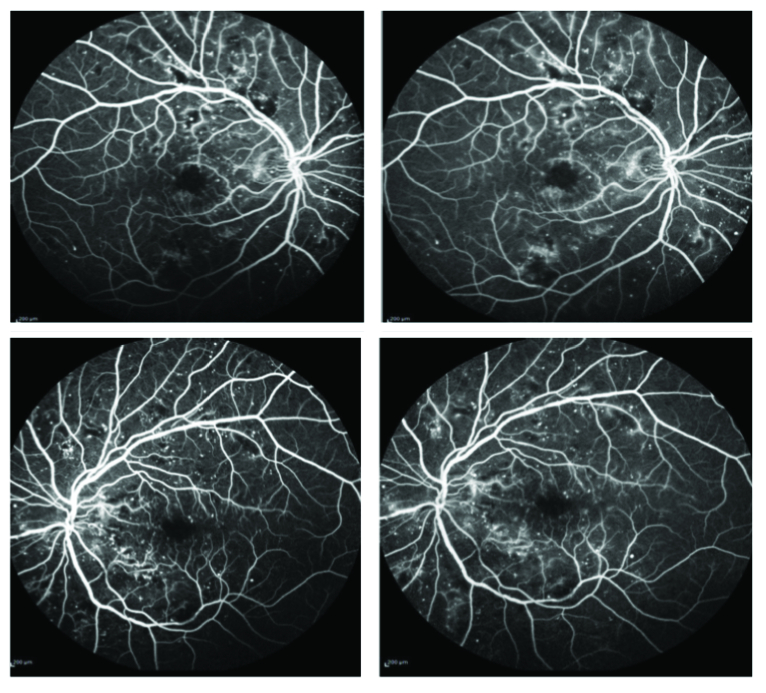
Fundus fluorescein angiography of the right (top left and right) and left (bottom left and right) eyes showing macular hypofluorescence, no vascular leakages and few areas of capillary non-perfusion.

**Figure 4:**
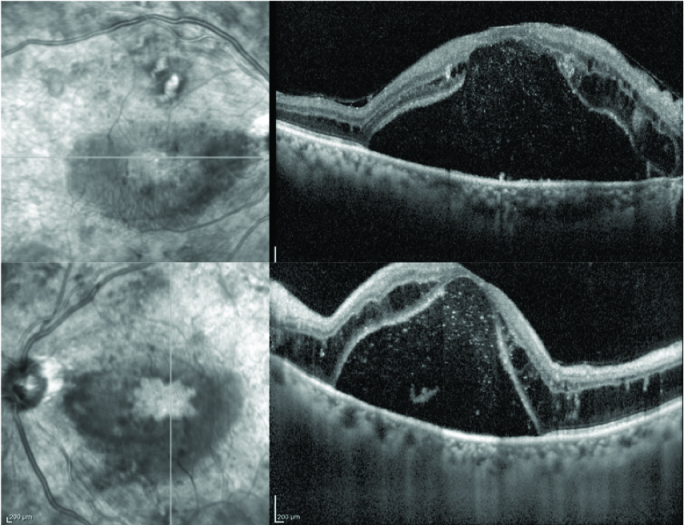
Optical coherence tomography of the right (above) and left (below) eyes showing worsening of cystoid macular edema and serous macular detachment despite treatment.

**Figure 5:**
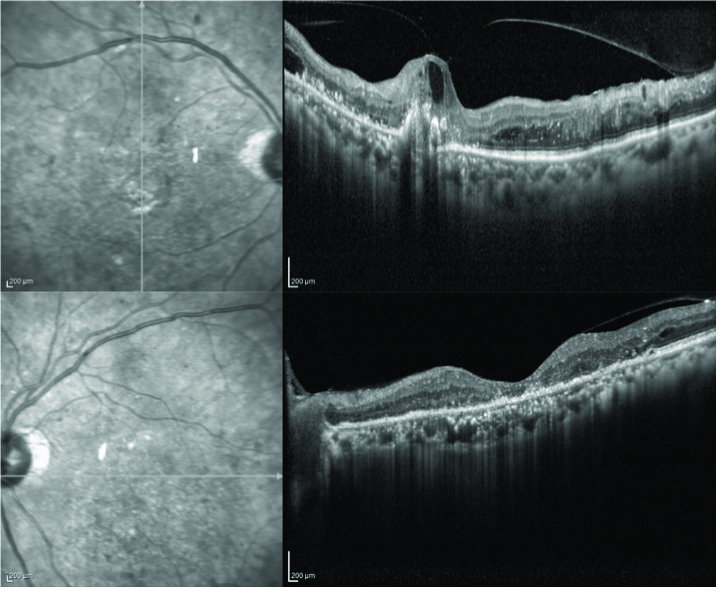
Optical coherence tomography of the right (above) and left (below) eyes following systemic treatment for multiple myeloma.

##  CASE REPORT

A 65-year-old Indian male complained of progressive blurring of vision in both eyes for three months with right-sided headache and shoulder pain. He has been a moderately controlled diabetic and hypertensive patient for 10 years. At the initial ophthalmologic examination, the corrected distance visual acuity (CDVA) was 20/120 in the right eye (RE) and 20/80 in the left eye (LE). The anterior segment examination was normal in both eyes. The dilated fundus examination revealed multiple dot and blot and flame-shaped hemorrhages in all quadrants with macular edema in both eyes [Figure 1]. The working diagnosis at this time was very severe non-proliferative diabetic retinopathy (NPDR) with DME.^[6]^

Spectral domain OCT (SDOCT; Spectralis, Heidelberg Engineering, Heidelberg, Germany) in both eyes revealed CME and SMD with a central foveal thickness (CFT) of 527 microns in the RE and 628 microns in the LE [Figure 2]. Fundus fluorescein angiography revealed a normal arm-to-retina time, macular hypofluorescence, no vascular leakages, and few areas of capillary non-perfusion [Figure 3]. Over the following nine months, the patient received three bevacizumab (1.25 mg/0.05 ml) and two triamcinolone acetonide (4 mg/0.1 ml) intravitreal injections in each eye. There was some, though never complete, resolution of the subretinal fluid in both eyes during this period; however, the edema showed no response to the treatment and there was an increase in subretinal hyperreflective foci. The patient was lost to follow-up for four months and returned with a CFT of 953 microns and 1122 microns in the RE and LE, respectively [Figure 4]. After systemic evaluation, the diabetes appeared to be fairly well-controlled. The patient was referred to a hematologist to rule out other underlying systemic pathologies and was diagnosed with multiple myeloma after a detailed evaluation.

The patient was under the care of a hemato-oncologist for about a year during which he did not undergo any ophthalmic treatment. During remission 13 months later, he came to us with the complaint of further decrease in vision-to-hand movements in both eyes. The examination revealed that there was a mature cataract in both eyes for which he underwent phacoemulsification surgery with intraocular lens implantation one month apart for each eye. Post-surgery, the CDVA was 20/80 in the RE and 20/60 in the LE. The fundus examination in both eyes showed multiple dot and blot and flame-shaped hemorrhages with a decreased SMD and hard exudate plaque present at the fovea. The SDOCT of the RE showed vitreomacular adhesion, minimal subretinal fluid, and subfoveal ellipsoid zone (EZ) disruption with a CFT of 340 microns and the LE showed complete resolution of the SMD and EZ disruption with a CFT of 265 microns [Figure 5]. In the last follow-up exam, vision further improved to 20/60 in the RE and 20/30 in the LE, with the fundus picture and SD-OCT findings remaining stable.

##  DISCUSSION

Multiple myeloma is a plasma cell malignancy that damages renal, skeletal, and neurological functions.Deposition of immunoprotein in the cornea and pars plana, and IgM deposits in all layers of the retina is also observed.^[7]^ Paraproteinemia, or monoclonal gammopathy, is the presence of excessive amounts of a single monoclonal gammaglobulin (or paraprotein) in the blood.^[6]^ An unusual SMD with or without subretinal precipitates or fundus signs of serum hyperviscosity, such as retinal hemorrhages and distended retinal veins, may be observed in patients with hypergammaglobulinemia and immunogammopathy such as systemic lupus erythematosus, Waldenstrom's macroglobulinemia, and multiple myeloma.^[8,9,10]^

While paraproteinemic maculopathy is typically bilateral due to the underlying systemic pathology, there have been reports of unilateral involvement.^[7]^ Hyperviscosity, secondary to systemic illnesses, can cause stasis and hypoxia of the endothelial cells and weaken the blood–retinal barrier.^[7]^ This increases the deposition of immunoglobulins in the subretinal space and creates a higher osmotic gradient leading to subretinal fluid accumulation.^[11]^ Other risk factors for retinal vascular endothelial damage and ischemic injury like anemia, diabetes, and hypertension can coexist in these patients and cause worsening of the leakage and breakdown of the blood–retinal barrier. Diabetic retinopathy, involving the macula, can be particularly confounding due to a similar morphology. Adult vitelliform dystrophy and central serous chorioretinopathy can also mimic paraproteinemic maculopathy.^[7]^

Resolution of the SMD in paraproteinemic maculopathy requires treatment of the underlying hematological pathology, albeit the response may be slow or incomplete. Decreasing the blood immunoglobulin level is the primary goal with plasmapheresis, systemic corticosteroids, or systemic chemotherapy.^[12]^ After administering ocular treatment with intravitreal anti-vascular endothelial growth factor and steroid injections, the affected eyes do not show much response due to underlying systemic immunogammopathy.^[7,8,13]^

In summary, this case demonstrates that for patients who suffer from non-resolving or atypical macular edema and are resistant to conventional treatment, it is imperative to consider the possibility of underlying systemic pathologies. One needs to determine the existence of other vascular features, morphology of the hemorrhages, and/or the presence of a SMD.^[14]^ Patients who may present with particular ocular symptoms at first should be further screened resulting in an early referral, diagnosis, and treatment which may be lifesaving.

##  Declaration of Patient Consent 

A written informed consent was obtained from the patient for publication of this case report and any accompanying image/images. A copy of the written consent is available for review by the Editor-in-Chief of this journal.

##  Financial Support and Sponsorship

None.

##  Conflicts of Interest

None.
